# Ultra-low dose immunoPET using ^64^Cu-rituximab tracer for a human CD20 mouse model

**DOI:** 10.3389/fmed.2025.1548132

**Published:** 2025-04-07

**Authors:** Frezghi Habte, Arutselvan Natarajan

**Affiliations:** Department of Radiology, Stanford School of Medicine, Stanford University, Stanford, CA, United States

**Keywords:** immunoPET, ultra low dose, PET/CT imaging, human CD20, dosimetry

## Abstract

Antibodies (Abs) and their fragments can be labeled with PET radioisotope (immunoPET) for *in vivo* diagnostic imaging. Compared to the conventional FDG-PET, immunoPET can be designed to target *in vivo* cancer-specific antigen expression levels for various tumors and metastasis, which makes immunoPET (iPET) a powerful technique for molecular imaging and therapy monitoring. However, achieving the optimal dose to minimize radioisotope toxicity without compromising the visualization of the smallest tumor is challenging. To find an ultra-minimal tracer dose, we have developed a novel iPET with an intact rituximab Ab labeled with ^64^Cu to image human CD20 (hCD20) in a transgenic mouse model for non-Hodgkin’s lymphoma (NHL) imaging. Using phantom and *in vivo* mouse models, we optimized the minimal dose that can be administered in a mouse using a high-specific iPET tracer prepared from ^64^Cu-rituximab. A phantom study was used to characterize the scanner capability and limit for imaging using low doses. An ultra-minimal dose administered in a mouse model showed good image quality with high signal-to-noise ratio without compromising quantitative accuracy. The phantom study with below 50 μCi dose level indicated a slight increase in variability due to reduced dose specifically for target regions with lower uptakes (<3:1 ratio) relative to the background. *In vivo* study performed with four groups of mice (*n* = 3), each group injected with ~90, ~50, ~25, and ~10 μCi showed a linear increase of tracer uptake measured as percentage injected dose per gram (%ID/g). This tracer has shown high specific uptake in the spleen, where most B-cells are engineered to express hCD20. The study demonstrated that the lowest dose threshold limit for ^64^Cu-antibody-based iPET was about 25 μCi while achieving a high-quality image and quantitative accuracy.

## 1 Introduction

Immuno-positron emission tomography (immunoPET) a.k.a. antibody-based PET molecular imaging strategy is performed taking advantage of the high specificity of monoclonal antibody (mAb) and the inherent high sensitivity of PET (1–10). Several radionuclides and mAbs have been exploited to develop immunoPET (iPET) probes, some of which have already been successfully translated for clinical use (4, 7, 8, 11–13). iPET is becoming the method of choice for imaging specific tumor markers, immune cells, immune checkpoints, inflammatory processes, and guide mAb-based therapy (12, 14–18). Superior to conventional FDG-PET, immunoPET can characterize and quantify antigen expression specific to a tumor type, making iPET a powerful molecular strategy for tracking, visualizing, and measuring the tumor gene expression (18–24). FDG-PET, taken up into the body through glucose transporters, has very poor specificity and can be seen in areas with high levels of metabolism and glycolysis, such as sites with inflammation or tissue repair (23–28). iPET has the potential to image specific diseases and quantify them for clinical diagnostic applications. Several iPETs are already in clinical investigations for cancer staging and therapy monitoring using FDA-approved mAbs (4, 29). We have recently developed a novel iPET tracer using mAb (rituximab) labeled with ^64^Cu and ^89^Zr to image human CD20 as a marker for NHL. Evaluation of these new tracers in a transgenic mouse model and humans showed specific imaging of hCD20-expressing B-cells (30–33). Such new iPET tracers in development have immense potential in the clinical setting as the antibody can bring radiation directly to the lymphoma cells (34–38).

When compared to FDG-PET, the iPET is linked with long-half-life radioisotopes to match the slow uptake and clearance of antibodies selected for iPET imaging. As a result, the radiation burden on the patient when using antibody-based tracers is relatively high compared to conventional rapidly clearing PET tracers such as [^18^F]FDG (35, 36). This limitation could hinder the development and practical application of antibody-based tracers, and therefore, it is paramount to reduce the radiation exposure whenever feasible. Advances in iPET imaging uses modified antibody fragments with small size and shorter elimination half-life allowing the use of short half-life radioligands to perform iPET imaging on the same day while reducing radiation exposure (38–43).

Engineered smaller size affibody proteins and antibody fragments retaining the essential specificities and affinities of a full antibodies, have become desirable pharmacokinetics for PET imaging using various options of PET isotopes (3, 38, 40). While these new iPET imaging strategies will play a big role in reducing radiation exposure, we also believe that administration of an optimized minimum dose could also substantially decrease the overall radiation burden on the patient (43–45). However, since PET inherently produces noisy images, it is challenging to optimize the minimum dose (46–49). Reducing the injected dose amount may further compromise the image quality (44, 45). Since PET measures the biodistribution of a particular tracer administrated to the body, the number of detected tracers counts on a selected region of interest defines the image quality and quantitative accuracy. Hence, dose administration could be specific to each tracer behavior or condition of the study. This means, there is a limit and variation to the minimum dose required that provides adequate counts for obtaining a non-biased signal-to-noise ratio and quantitative diagnostic value specific to each study (45). To evaluate this more quantitatively, in this study we evaluated the ultra-low injected dosage capability of iPET using phantom and live animal models. The combined effect of the high sensitivity of PET/CT and the high specificity of iPET tracers (32, 43) may allow the administration of ultra-low doses without compromising the image quality and quantitative accuracy.

## 2 Materials and methods

### 2.1 iPET tracer and animal model

GMP grade ^64^Cu labeled iPET tracer preparation was already reported (33, 50). To test the tracer capability for low-dose diagnostic imaging, we have used a transgenic mouse model that expresses hCD20. In this mouse model, hCD20 is expressed in B cells homing in the spleen, providing the highest tracer uptake. For this tracer development, the anti-hCD20 antibody (IgG; rituximab) was conjugated to DOTA for radiolabeling of ^64^Cu. In another study, we have reported the evaluation of the dosimetry of ^64^Cu-mAb tracer in the hNSG mouse model using the standard average injected dose of 100 μCi (32).

### 2.2 Phantom study

**Figure 1:**
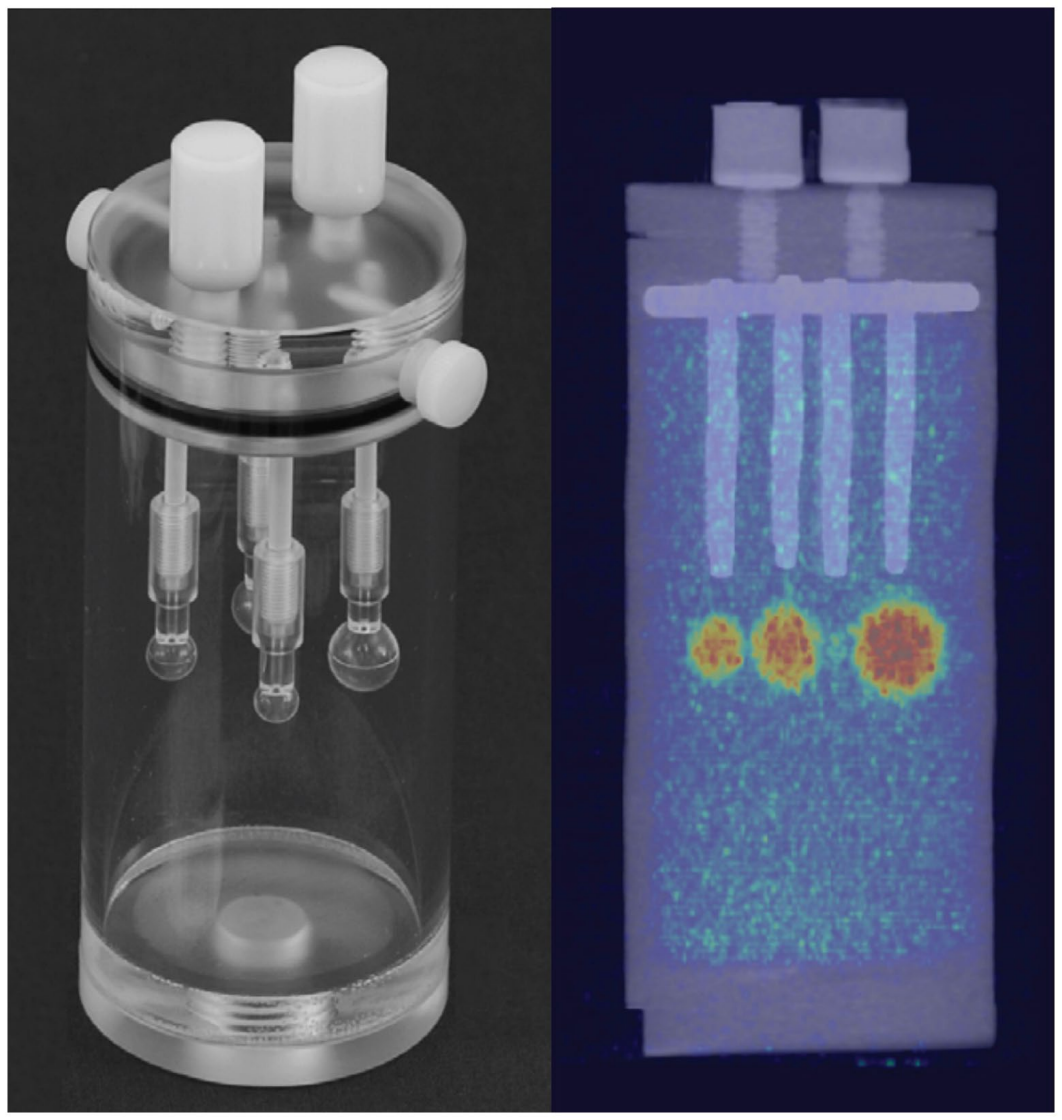
Left, Cylindrical tube with four insertable fillable hollow spheres of different sizes (~1, 0.5, 0.25, and 0.031 ml) used as a phantom. Right, PET/CT image of phantom after filling the cylinder tube with low radiotracer concentration (~1 μCi/cc) as background and hollow spheres with ~7.5 μCi/cc as foreground uptake.

To evaluate the ultra-low dose counting accuracy of the scanner, we used a cylindrical phantom (Data Spectrum Corp) with a 40 mm inner diameter, 82 mm height, and four micro-hollow fillable spheres (Figure 1). The cylindrical phantom was filled with water and ~95 μCi of ^64^Cu to provide ~1 μCi/cc radiotracer concentration as a background signal for the phantom study. To represent the foreground signal and emulate high tracer uptake in an animal model, we prepared a 20 ml methanol and ~150 μCi of ^64^Cu solution. Due to its lower density relative to water, methanol was used as a contrast agent to provide slight CT image contrast in the PET/CT imaging. The mix provided ~7.5 μCi/cc ^64^Cu tracer concentration to fill each hollow four spheres (~1, 0.5, 0.25, and 0.031 ml). For the first scan, the total initial activity of the background and foreground was ~92 μCi. The Phantom was scanned using Inveon MicroPET/CT (Preclinical Solutions; Siemens Healthcare Molecular Imaging, Knoxville, TN) for 30 min at different time points while decaying over 48 h. Three image frames of 5, 10, and 20 min from the acquired images were reconstructed using the Ordered-subset expectation maximization (OSEM 2D) algorithm (51). Inveon Research Workspace (IRW) analysis software (Siemens Healthcare) was used for analysis and quantitation. Regions of Interest (ROI) were drawn over the four spheres and the background to obtain the mean radiotracer distribution (μCi/cc). Doses at each scan time were calculated from the decayed ^64^Cu tracer.

### 2.3 Animal study

Animal studies were performed in compliance with approval from the Administrative Panel on Laboratory Animal Care (APLAC) at Stanford University. The hCD20 transgenic mice models (Genentech, South San Francisco, CA) were used for the experiment. Prior to the animal study, transgenic mice were screened by RT-PCR to confirm the expression of hCD20 positivity. We injected 8–10, 20–25, 45–55, and 75–80 μCi via the tail vein in a group of four mice for each dose. After radiotracer administration, the animals were imaged at ~1–5, 15, and 24 h using Inveon MicroPET/CT within 20–30 min intervals.

### 2.4 PET imaging and analysis

PET imaging was performed on the Inveon MicroPET/CT system following standard routine acquisition protocol in our facility. The CT scan was acquired using an 80 kVp and 500 μA, two-bed position, half scan 220° of rotation, and 120 projections per bed position for both anatomic reference and PET attenuation correction. PET scans were performed using the default settings of a coincidence timing window of 3.4 ns and an energy window of 350 to 650 keV. Static 5 min was used to acquire first-time point acquisitions (1–5 h post-injection), followed by static 10 min acquisition for later time points (15 and 24 h). The images were reconstructed using the OSEM 2D algorithm. Using IRW, manual three-dimensional regions of interest (ROIs) were drawn over the heart, liver, and leg muscles. A semi-automatic ROI histogram-based segmentation technique was used to segment the spleen to reduce reader variability. The average radioactivity concentration in the ROI was obtained from the mean pixel values within the ROI volume, which is converted to a percentage injected dose per gram of tissue (%ID/g).

### 2.5 Statistical analysis

The quantitative data were expressed as mean ± SD. Means were compared using the student *t* test. A 95% confidence level was chosen to determine the significance between groups, with *p* values of less than 0.05 indicating significant differences.

## 3 Results

### 3.1 Phantom study

**Figure 2:**
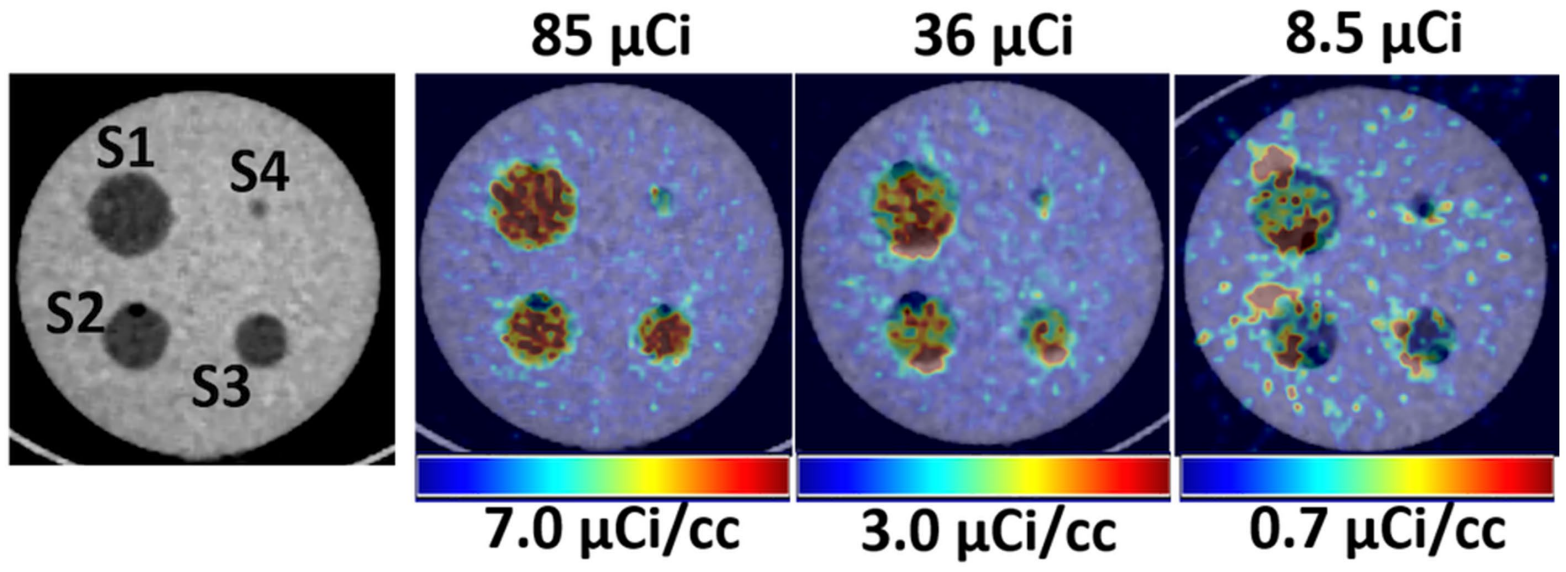
Left, CT images showing the hollow fillable spheres of the phantom. Right, Qualitative comparison of PET/CT images of the phantom for selected three low doses.

**Figure 3:**
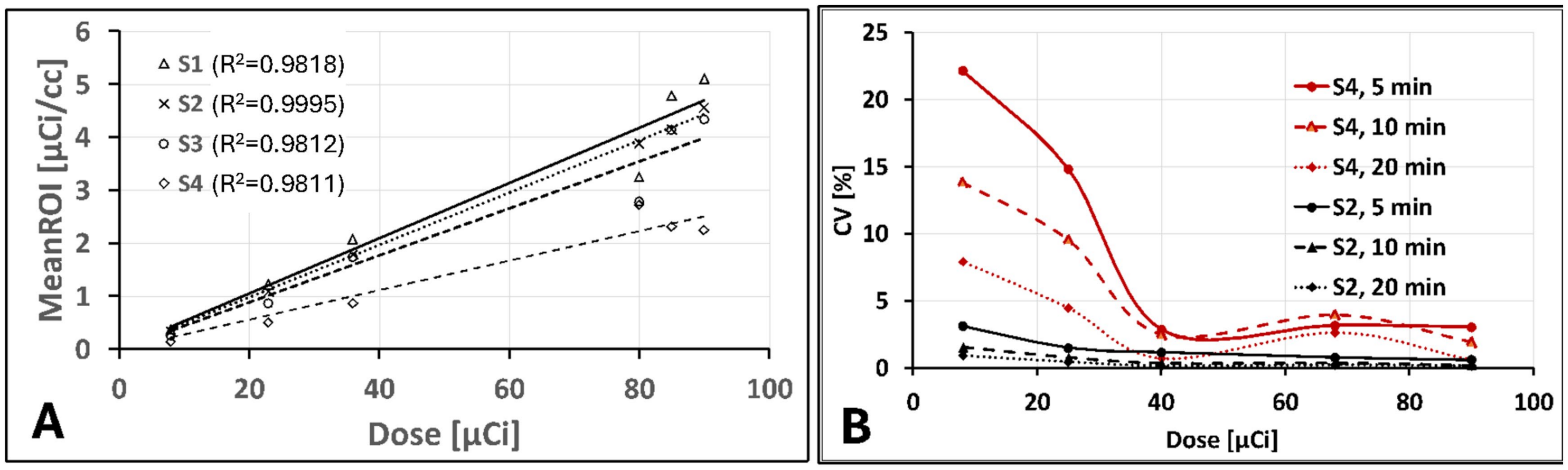
Phantom images quantitative assessments: **(A)** extracted mean ROI value vs. administered dose showing an expected linear increase with lower slop for smaller size spheres due to partial volume effect and **(B)** Computed percentage coefficient of variation showing only for S2 (second to largest) and S4 (smallest) sphere sizes for 5, 10, 20 min acquisition time as function of administrated dose.

Figure 2 shows a qualitative comparison of phantom images between conventional and ultra-low doses at scan time. After a suitable image intensity adjustment, the effect of low ultra-low dose <10 μCi shows nonhomogeneous uptake in all spheres. However, all hollow spheres, including the smallest (0.031 ml, S4), which shows the lowest uptake due to the partial volume effect, showed higher uptake than the background. Figure 3 quantitatively assesses the mean values extracted from ROIs drawn over each hollow sphere based on the CT images. The computed mean ROI vs. dose at scan time showed comparable linearity with the expected partial volume effect (Figure 3A). For comparison, we also computed the coefficient of variance of images reconstructed at different times of acquisition (5, 10, and 20 min) for each dose (Figure 3B, showing only the S2 (second largest) and S4 (smallest) sphere sizes). The result indicates that the computed variability increases slightly for doses less than 40 μCi for larger spheres independent of scan duration with expected improvement for images acquired with longer acquisition time (10 and 20 min).

### 3.2 Animal study

**Figure 4:**
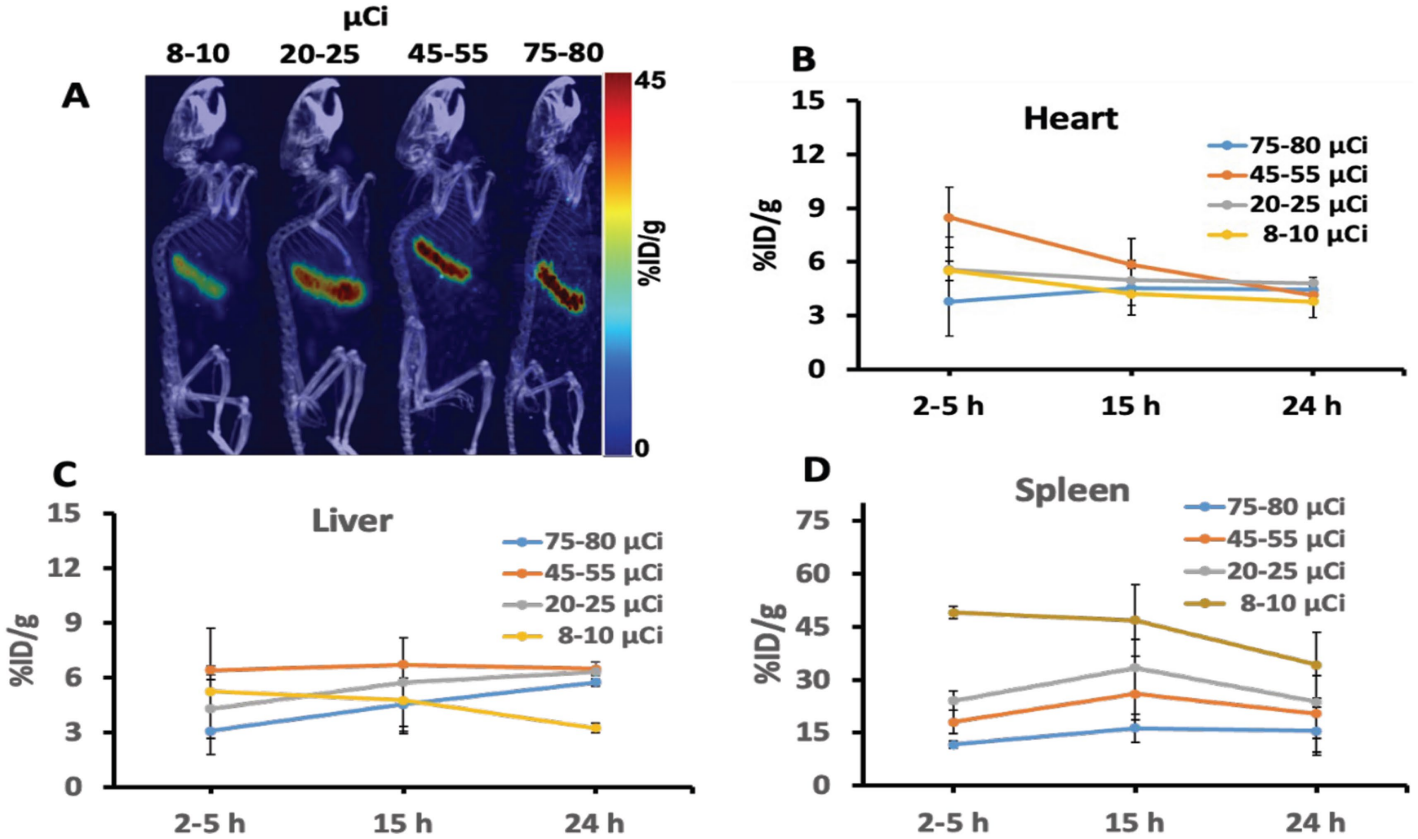
**(A)** Representative iPET/CT images showing tracer uptake 24 hr post injection in the spleen of the humanized transgenic mice. Each mice group received doses (ranging from 8 to 80 μCi). **(B–D)** Depicted the iPET signal corresponding to the tracer uptake from the heart, liver, and spleen of the mouse at different time points.

*In vivo*, animal imaging using the target-specific ^64^Cu iPET radiotracer, as expected, showed high uptake in the spleen for all dose amounts (Figure 4A). Relatively low uptake was also seen in the heart and liver. The spleen consistently showed increased uptake in %ID/g of 3 to 10 folds relative to the liver with a decreased injected dose (Figure 4D), showing improved tracer specificity and image contrast. In contrast, the spleen-to-liver uptake ratio steadily increased (3–5 folds) with a decrease of dose up to 20 μCi. The spleen-to-liver uptake increased non-linearly over nine-fold for ultra-low injected dose <10 μCi (*p* < 0.03). At 15 h post-injection (p.i.), high tracer uptake in the spleen was obtained (Figure 4D).

**Figure 5:**
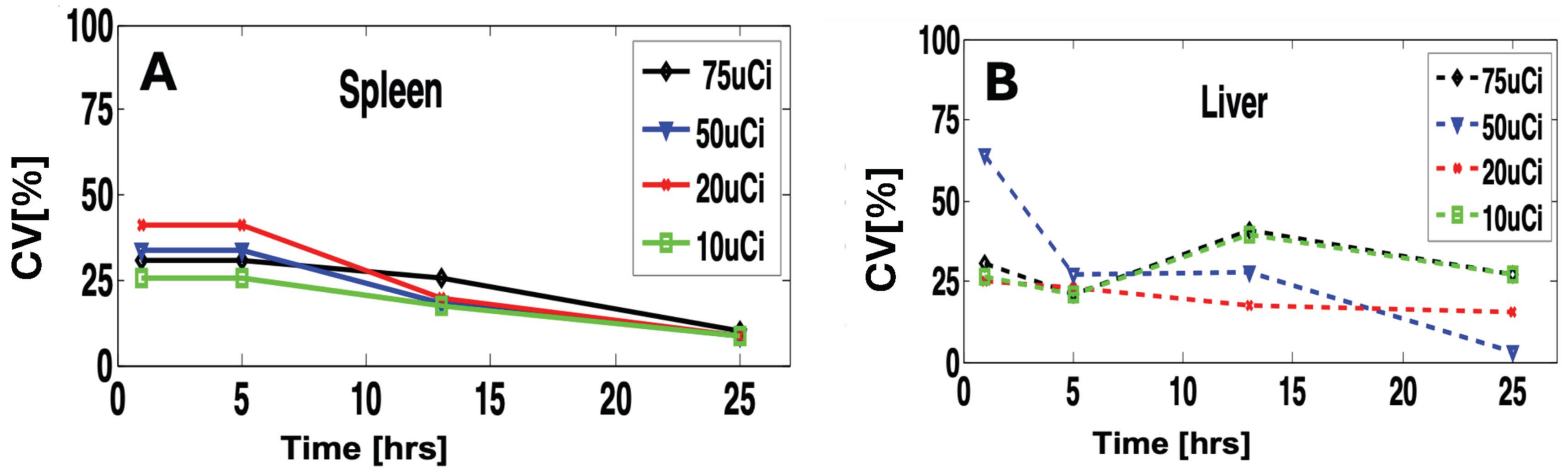
Quantitative comparison of variability within different doses and scan time. **(A)** for the spleen and **(B)** for the liver.

Figure 5 shows a quantitative assessment of inter-subject variability. For the spleen, we observed similar inter-subject variability with slight variation between doses (Figure 5A) and slightly higher variability (CV = ~30%) at the early time points (1 to 5 h). At the later time point (24 h.), the variability drops on average to one-third (CV = ~10%) due to tracer clearance (Figure 5B).

## 4 Discussion

As the most popular molecular imaging tool, PET/CT imaging has always been attractive for both preclinical and clinical applications due to its superior sensitivity. However, the inability to accurately determine the optimum dose that may be administrated to the subject may make to operate non optimally especially regarding minimization of radiation exposure to the subject and associated cost of the target tracer (43, 45). Specifically, the challenge is to find a standard minimum dosage (SMD) that is sufficient to acquire clinically relevant diagnostic information, which inherently involves multiple factors. Assuming a fixed scanner sensitivity limited by its specific geometric configuration and detector characteristics, the optimum minimum dosage may vary on several image acquisition parameters such as reconstruction type, subject size and positioning, and scanner acquisition protocols (43). Within the selected optimum acquisition and reconstruction parameters, dosage may also vary with the specific choice of radiopharmaceutical and its associated factors, including the tracer uptake specificity, clearance pattern, molecular size of the tracer, tracer half-life, and others.

Imaging of iPET using mAb labeled with Cu-64 or Zr-89 isotope provides relatively high specificity compared to the non-specific tracer such as FDG. This is because mAbs are specifically designed to bind to targeted organs or tumors selectively. Hence, a small tracer dose (26, 32) could specifically bind to the intended *in vivo* target organ or receptors. Using phantom and animal studies, our results indicated that as low as 20 μCi of tracer dose can be sufficient to image using the most commonly available MicroPET/CT scanners without significantly affecting or compromising image quality and quantitative value. Furthermore, iPET animal imaging has shown better image contrast with reduced background noise at lower doses compared to conventionally administered doses (~100 μCi per mouse). The overall results (Figure 4) indicate that an optimal suitable lower dose may improve image quality and quantitative accuracy while reducing radiation exposure. It is also expected to improve the image quality at lower doses by extending the scanning time (Figure 3B).

In the animal study, the spleen express hCD20 marker for NHL, showing the highest uptake of iPET tracer which is consistent as reported elsewhere (33, 50). This gives a promising application for tracing and tracking the metabolic activity in tumors expressing antigen CD20. This tracer has been specifically developed to target B-lymphocytes expressing hCD20 seen in the spleen with increased numbers in non-Hodgkin’s Lymphoma (32). It was anticipated to accumulate in the spleen with some lower uptakes in the heart and liver due to their roles in removing toxins from the body. If the tracer uptake in the spleen saturates, the extra tracer clears through the liver. Hence, the increased amount of tracer doses tends to bind on other organs non-specifically, such as the uptake seen in the liver (Figure 4C). On the other hand, lower administrated doses show to decrease non-specific organs uptake while increasing target-specificity. Spleen-to-liver ratios demonstrated distinctively that even at the lowest doses (<8 μCi) and after 24 h of decay, the spleen shows strong uptake with relatively good image quality. An estimated dose of 20 to 25 μCi, as reported in this study, could be assumed as the lower dose limit that may provide comparable image quality and accuracy relative to the commonly used higher doses for *in vivo* mouse iPET imaging. This study provides initial validation for the important and challenging tradeoffs in PET imaging between image quality and radiation exposure. The study demonstrates that with increased specificity of specific tracer a significantly lower dose up to one fourth as estimated in Figure 4 relative to conventional dose (~100 μCi) may be used without changes of the imaging protocols and compromising the image quality. Minimizing radiation exposure reduces the risks associated with ionizing radiation and the overall imaging costs, but it is also very important to have good image quality practically for all clinical and research applications (47, 48). We also expect this study to provide a bases for further validation of the optimum minimum dose for clinical practice, which is relatively more important due to the ever-increasing concerns related to radiation exposure in patients requiring multiple examinations or those at a higher lifetime risk for developing cancer (e.g., pediatric patients) (49).

## 5 Conclusion

Our study provides the basis for the initial validation of the potential usage of ultra-low-dose clinical practices using target-specific iPET imaging studies without impacting the overall image quality and quantitative accuracy. Low doses may also improve specificity and reduce radiation in non-targeted areas and non-specific uptake by other clearing organs such as the liver and kidney. With the evolving research on early cancer detection and immunotherapies, imaging with more targeted tracers will help reduce misdiagnoses and unnecessary radiation exposure. This study reveals that optimal low-dose estimation is vital to all diagnostic imaging tracers prior to clinical translation studies, saving tracer costs and reducing systemic radiation exposure.

## Data Availability

The raw data supporting the conclusions of this article will be made available by the authors, without undue reservation.
