# Supplementary figures and images for: Ultra-low dose immunoPET using 64Cu-rituximab tracer for a human CD20 mouse model
Source: Front Med (Lausanne). 2025 Apr 7;12:1548132. doi: 10.3389/fmed.2025.1548132 (PMC12010902; doi:10.3389/fmed.2025.1548132)

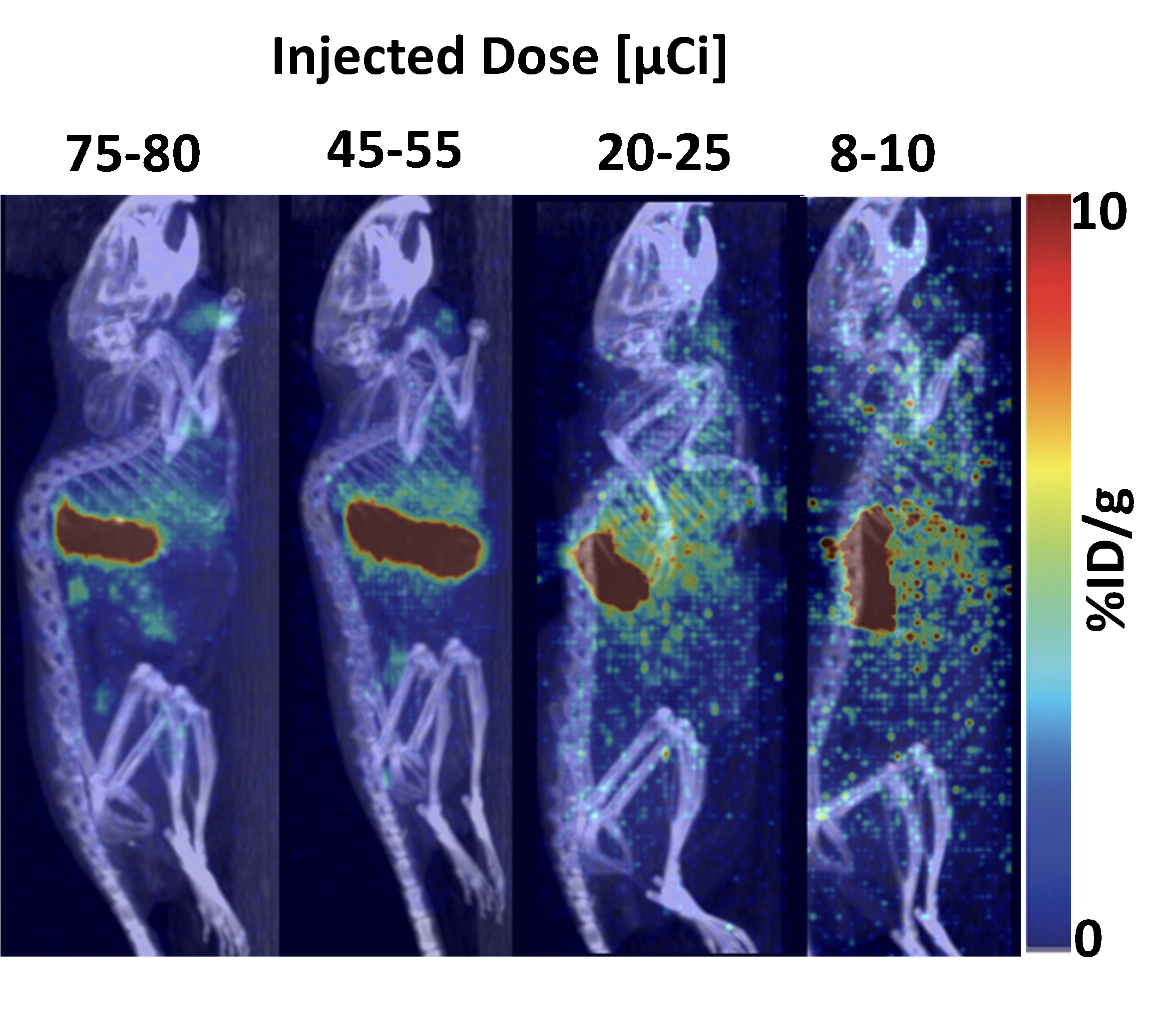

Supplement: Supplementary file 1 [file Image_1.tiff]
